# The effect of mild to moderate COVID-19 infection on the cardiorespiratory fitness of firefighters

**DOI:** 10.3389/fpubh.2023.1308605

**Published:** 2023-11-30

**Authors:** Susanne D'Isabel, Lauren M. Berny, Alex Frost, Chanhtel Thongphok, Kepra Jack, Sundeep Chaudhry, Ross Arena, Denise L. Smith

**Affiliations:** ^1^First Responder Health and Safety Laboratory, Department of Health and Human Physiological Sciences, Skidmore College, Saratoga Springs, NY, United States; ^2^Department of Counseling Psychology and Human Services, University of Oregon, Eugene, OR, United States; ^3^Department of Health and Human Physiological Sciences, Skidmore College, Saratoga Springs, NY, United States; ^4^HeartFit for Duty, Mesa, AZ, United States; ^5^MET-Test, Atlanta, GA, United States; ^6^Department of Physical Therapy, University of Illinois at Chicago, Chicago, IL, United States

**Keywords:** cardiorespiratory fitness (CRF), firefighters, firefighting, COVID-19, cardiopulmonary exercise testing (CPET)

## Abstract

**Introduction:**

An adequate level of cardiorespiratory fitness (CRF) is critical for firefighters to perform the strenuous and physiologically demanding work of firefighting safely and effectively. The coronavirus disease 2019 (COVID-19) has been shown to negatively impact CRF in both the acute phase and longer-term following infection. This study aimed to determine changes to the CRF of firefighters pre- to post-mild to moderate COVID-19 infection and to investigate the impact of days past COVID-19 infection on change in CRF.

**Methods:**

CRF measures from cardiopulmonary exercise testing (CPET) at annual occupational health exams that occurred pre-COVID-19 infection in 2019 were obtained for firefighters from seven Arizona fire departments. Measures were compared to CPET evaluations from annual health exams the following year in a cohort of firefighters who self-reported mild to moderate illness following COVID-19 infection between exams.

**Results:**

Among a cohort of 103 firefighters, mean age 40 ± 9 years, CRF [as measured by peak oxygen consumption (VO_2_)] declined by an average of 2.55 ml·kg^−1^·min^−1^ or 7.3% (*d* = −0.38, *p* < 0.001) following COVID-19 infection (mean time from COVID-19 infection to CPET was 110 ± 78 days). The number of days past COVID-19 infection showed a small, yet significant, relationship to peak VO_2_ (*r* = 0.250, *p* = 0.011). Estimated marginal effects indicated that when biological sex, age, and BMI are controlled for, predicted peak VO_2_ returned to pre-COVID-19 values ~300 days after COVID-19 infection.

**Conclusion:**

Peak VO_2_ (ml·kg^−1^·min^−1^) declined 7.3% among firefighters an average of 110 days past reporting mild to moderate COVID-19 infection. This decrease has implications for the operational readiness and safety of firefighters.

## 1 Introduction

Cardiorespiratory fitness (CRF) is an integrated measure of the cardiovascular and respiratory systems' ability to supply oxygen to the body to meet the metabolic demands of working skeletal muscle during maximal exertion. High levels of CRF are associated with significantly lower risk of cardiovascular disease, all-cause and cardiovascular disease mortality, and incidence and mortality from certain cancers (1–4). Adequate levels of CRF are particularly important in occupations such as firefighting where strenuous work tasks are performed under physiologically taxing conditions that can impose pronounced strain on the cardiovascular system (5–8). As sudden cardiac events are the leading cause of duty-related deaths for firefighters (9–11) and CRF is associated with individual cardiovascular disease risk factors and the overall cardiovascular risk profiles of firefighters (12, 13), identifying and better understanding factors that negatively affect firefighters' CRF is important for the fire service and clinicians who care for these public safety personnel. Given the vital mission firefighters perform, their health is also critical to the public they serve.

Recent research has presented convincing evidence that coronavirus disease 2019 (COVID-19) infection can result in decreased CRF (14–22). Although elapsed time since illness appears to mitigate CRF decrements (15, 17), reduced CRF can persist for months following COVID-19 infection (15, 17, 20–22). Unsurprisingly, CRF decrements are more pronounced in those who experienced severe illness (14, 17, 19, 22). Studies examining CRF changes following COVID-19 infection are, however, limited because the criterion measure of CRF, specifically cardiopulmonary exercise testing (CPET), is not routinely administered to the general population. Consequently, most published studies use an observational cohort design comparing findings post-COVID-19 to an uninfected control group, historical controls, or population-based norms. Studies were also primarily conducted among hospitalized subjects or those experiencing long-term symptoms, limiting generalizability to those who had mild or moderate illness and resolved symptomology. Better understanding the change in CRF of individuals who had mild or moderate COVID-19 illness is particularly relevant as this group represents the vast majority of COVID-19 cases (23).

Due to limited use of CPET, few studies have been able to employ a longitudinal, single group design to examine pre- and post-COVID-19 infection changes in CRF, particularly with participants who experienced mild to moderate symptoms. Studies that have, showed significant declines in CRF from pre- to post-COVID-19 infection (21, 24, 25). Generalizability of findings to the fire service, however, is limited due to study participant characteristics being unlike the fire service. One study of healthcare workers consisted primarily of older women (21) and two additional studies utilized data from competitive athletes—a uniquely fit group (24, 25).

Despite the importance of CRF in firefighting, we are unaware of previous investigations examining the effects of COVID-19 on the CRF of firefighters. Such an investigation is warranted as any decrement in CRF could pose a serious threat to firefighter health and safety, and thus could impair performance of their public safety mission. Therefore, the purpose of this study was to (1) examine CRF changes in a sample of active, career firefighters who completed an annual occupational medical exam that included assessment of CRF via CPET before and after mild to moderate COVID-19 infection; and (2) identify whether elapsed time since COVID-19 diagnosis was a significant predictor of change in CRF.

## 2 Methods

### 2.1 Study design

Data from 2019 annual occupational medical evaluations were used as pre-COVID-19 measures and data from 2020 annual medical evaluations were used as post-COVID-19 measures in a cohort of firefighters who reported that they had COVID-19 between the medical evaluations. Annual medical evaluations were conducted based on regular departmental testing schedules, thus the time between the onset of COVID-19 and subsequent CPET evaluations varied among individuals based on when they experienced COVID-19 infection. Days since onset of COVID-19 was reported by individual firefighters.

### 2.2 Study population

A total of 152 firefighters reported to the occupational health clinic on a supplemental COVID-19 survey that they experienced COVID-19 between February 2020 and February 2021. Records were excluded if the firefighter reported being hospitalized, or if data was not available for pre and post COVID-19 CPET. The final analytical sample consisted of 103 professional firefighters from seven Arizona fire departments. Information collected included date of diagnosis, testing, symptom onset, and questions about severity. Nine participants did not indicate a date of diagnosis; in these cases, date of testing or date of symptom onset was used. Firefighters from these departments receive yearly occupational medical evaluations from a single occupational healthcare clinic that measures CRF using CPET, considered the gold standard for assessing CRF (26). Firefighters were included in this study if they had a CPET evaluation at their 2019 medical evaluation (pre-COVID-19 measure), had a mild to moderate case of COVID-19 prior to their 2020 medical evaluation, and had CPET administered at their 2020 medical evaluation (post-COVID-19 measure), which could have occurred in the first 2 months of 2021 based on scheduling issues.

The study protocol was submitted to the Skidmore College Institutional Review Board and received a “Not Human Subjects Research” determination as researchers only interacted with de-identified data used for analysis.

### 2.3 Procedures

The annual incumbent medical evaluation was performed by a single occupational health clinic and was consistent with current guidelines for firefighting medical clearance (27). This clinic routinely assesses CRF using CPET on an upright cycle ergometer. A single provider trained by a cardiology team administered the tests at 2019 annual exams (pre-COVID-19) and 2020 annual exams (post-COVID-19). Resting lung function, including forced vital capacity (FVC), forced expiratory volume in the first second (FEV_1_), and the percent of lung capacity able to be exhaled in one second (FEV_1_/FVC) was assessed via spirometry prior to administering the CPET. Resting heart rate (HR) from ECG tracings and blood pressure (manual auscultation by a trained technician) were obtained prior to initiating the exercise protocol, and throughout the exercise test. CPET assessment included gas analysis that permitted the direct assessment of peak VO_2_, the primary dependent variable of interest, expressed as L·min^−1^, ml·kg^−1^·min^−1^, and percent predicted. CPET assessment also permitted the evaluation of additional variables that provide insight into potential limitations of peak VO_2_ including work rate (WR), work efficiency (ΔVO_2_/ΔWR), respiratory exchange ratio (RER), minute ventilation (V_E_), breathing reserve, peak O_2_ pulse (VO_2_/HR; index of stroke volume), HR/WR slope, O_2_ consumption at anaerobic threshold (AT), ventilatory efficiency slope (V_E_/VCO_2_ slope), and O_2_ uptake efficiency slope (OUES).

### 2.4 Data analysis

To address the study's first aim, we used paired *t*-tests to assess whether unadjusted differences in pre- and post-COVID-19 CRF parameters' means were statistically significant. As a measure of their magnitudes, standardized mean difference effect sizes were calculated with the Cohen's *d* formula specified for pre-post comparisons (28). To provide context on annual change prior to the COVID-19 pandemic, 2018 and 2019 peak VO_2_ measurements were also compared in a subset of firefighters in the study's analytic sample who had medical evaluations with CPET during both years.

In order to examine whether elapsed time since COVID-19 diagnosis was a significant predictor of change in CRF, we first created a peak VO_2_ change score variable by subtracting each individual's post-COVID-19 peak VO_2_ measurement from their pre-COVID-19 peak VO_2_ measurement such that a negative score indicates a reduction in CRF. Preliminary bivariate correlation analyses were conducted to assess the unadjusted linear relationships between peak VO_2_ change and days post-COVID-19 as well as four other control variables: pre-COVID-19 peak VO_2_ measurement, sex (0 = female, 1 = male), age (in years), and body mass index (BMI; kg·m^−2^) calculated from post-COVID-19 height and weight data. A linear regression model was fitted, with change in peak VO_2_ regressed onto the independent variables. To account for clustering of firefighters within departments (ICC = 0.034, design effect = 1.46) the model was estimated using cluster robust standard errors. Model performance was evaluated by confirming all linear regression assumptions were met. The model estimates were then used to plot the predicted change in peak VO_2_ across the range in elapsed time, holding the other continuous covariates constant at their average values and sex at male. All inferential analyses assessed statistical significance at α = 0.050.

## 3 Results

**Figure 1:**
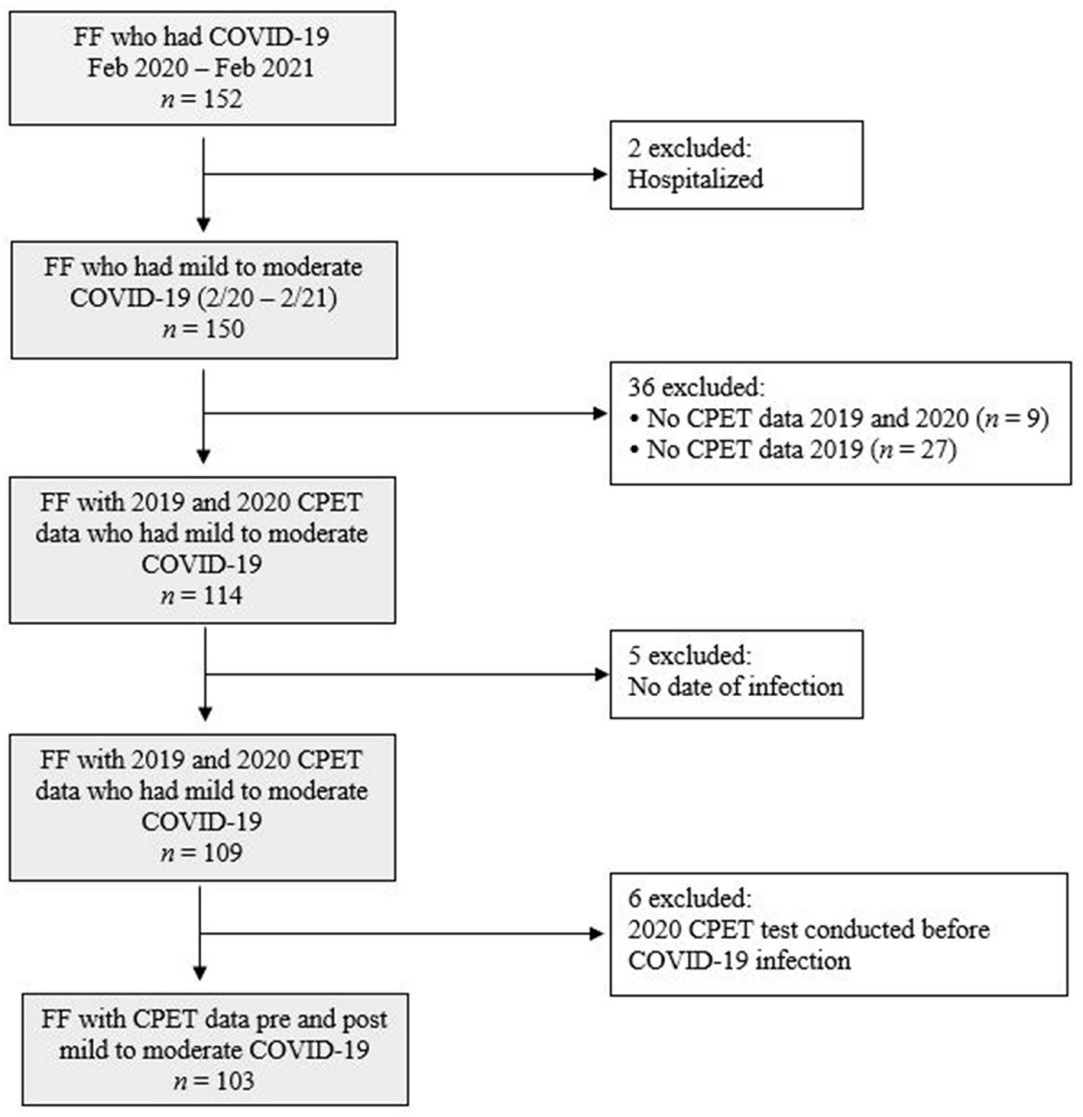
Record exclusion flowchart. FF, firefighter; CPET, cardiopulmonary exercise testing.

The record exclusion flow-chart is presented in Figure 1. In total, 152 firefighters reported that they had COVID-19 between February 2020 and February 2021. Ninety (87%) subjects self-reported testing information, of whom all indicated a positive test from PCR or antigen testing. A severe case of COVID-19 was defined as requiring hospitalization, thus two firefighters with self-reported COVID-19-related hospitalizations were excluded. By excluding these subjects, we were left with a sample whose illness ranged from asymptomatic (firefighters who reported no days with symptoms) to mild/moderate (firefighters who reported experiencing symptoms, but did not require hospitalization). To explore longitudinal changes in CRF pre- to post-COVID-19, 36 firefighters who did not have CPET data in both 2019 and 2020 were excluded from analysis. Six firefighters for whom a date of COVID-19 diagnosis could not be determined were also excluded. The final analytical sample consisted of 103 firefighters from seven departments who had CRF assessed via CPET prior to and after having mild to moderate COVID-19 infection.

Firefighter characteristics (post-COVID-19 timepoint) are presented in Table 1. The mean age of the firefighter cohort was 40.1 ± 9.3 years. Consistent with fire service demographics, only 8.7% of firefighters were female. The majority of the study cohort (83.5%) identified as White for their race/ethnicity and 12.6% identified as Hispanic. BMI (29.1 ± 4.0 kg·m^−2^) did not differ significantly from pre- to post-COVID-19 (*p* = 0.065). At the time of post-COVID-19 CPET, the mean number of days since firefighters had experienced COVID-19 was 109.7 (± 78.2) days.

**Table 1:** Firefighter characteristics (*n* = 103).

**Variable**	**Mean ±*SD* or *n* (%)**
Age (years)	40.1 ± 9.3
Sex (female)	9 (8.7%)
Race (white)	86 (83.5%)
BMI (kg·m^−2^)	29.1 ± 4.0
Time post-COVID-19 (days)	109.7 ± 78.2

Characteristics are presented for post-COVID-19 timepoint.

SD, standard deviation; BMI, body mass index.

Table 2 presents changes pre- to post-COVID-19 for vital signs, spirometry, and CPET parameters. Among the measured vital signs, resting systolic blood pressure (SBP) increased significantly (*d* = 0.50, *p* < 0.001), whereas no significant changes in resting diastolic blood pressure (DBP) and resting heart rate (HR) were observed. Similarly, there were no significant changes in spirometry measures (FVC, FEV1, and FEV1/FVC).

**Table 2:** Comparison of vital signs, spirometry and CPET parameters in firefighters pre- and post-COVID-19.

**Variables**	**Pre-COVID-19 *M* (*SD*)**	**Post-COVID-19 *M* (*SD*)**	**Difference**	**Cohen's *d***
**Vital signs**				
Resting HR (beats·min^−1^)	72.5 (11.5)	73.5 (11.3)	0.98	0.09
Resting SBP (mmHg)	118.7 (8.9)	123.1 (8.8)	4.44^**^	0.50
Resting DBP (mmHg)	78.9 (7.3)	79.3 (7.8)	0.37	0.05
**Spirometry**				
FVC (%)	97.6 (9.9)	97.5 (9.7)	−0.17	−0.02
FEV_1_ (%)	96.9 (10.3)	97.2 (10.7)	0.24	0.02
FEV_1_/FVC	79.6 (5.2)	79.7 (5.2)	0.08	0.02
**Exercise performance**				
Peak VO_2_ (L·min^−1^)	3.2 (0.6)	3.0 (0.6)	−0.20^**^	−0.32
Peak VO_2_ (ml·kg^−1^·min^−1^)	34.9 (6.7)	32.4 (6.7)	−2.55^**^	−0.38
Percent predicted	110.1 (16.9)	104.2 (16.2)	−5.89^**^	−0.36
**Work/exertion**				
Peak WR (watts)	258.2 (48.3)	256.8 (49.7)	−1.41	−0.03
Work efficiency (ΔVO_2_/ΔWR)	10.4 (1.1)	9.9 (1.7)	−0.50^*^	−0.34
Peak RER	1.06 (0.07)	1.07 (0.07)	0.01	0.08
**Pulmonary**				
Peak V_E_ (L·min^−1^)	105.6 (20.8)	105.4 (20.1)	−0.16	−0.01
Breathing reserve	38.2 (12.8)	36.7 (11.6)	−1.51	−0.12
**Cardiovascular**				
HR peak (beats·min^−1^)	161.1 (13.1)	161.7 (14.6)	0.63	0.05
SBP peak (mmHg)	168.3 (16.2)	157.4 (14.6)	−10.93^**^	−0.71
DBP peak (mmHg)	94.0 (7.8)	91.1 (6.0)	−2.91^**^	−0.42
Peak O_2_ pulse (ml·beat^−1^)	20.0 (3.9)	18.7 (4.1)	−1.29^**^	−0.32
Percent predicted	124.0 (18.6)	116.4 (20.1)	−7.54^**^	−0.39
HR recovery (beats·min^−1^)	88.6 (14.9)	88.3 (14.8)	−0.31	−0.02
HR/WR slope	0.36 (0.59)	0.44 (0.88)	0.08	0.10
**Anaerobic threshold**				
Anaerobic threshold (ml·kg^−1^·min^−1^)	17.5 (4.2)	13.3 (3.2)	−4.27^**^	−1.12
Percent peak VO_2_	55.4 (12.2)	42.9 (10.1)	−12.45^**^	−1.10
HR at anaerobic threshold	106.6 (12.5)	100.4 (11.6)	−6.19^**^	−0.51
**Ventilatory efficiency**				
V_E_/VCO_2_ slope	24.7 (3.3)	26.0 (3.3)	1.30^**^	0.39
OUES	3.11 (0.61)	2.93 (0.60)	−0.18^**^	−0.30

*M*, mean; *SD*, standard deviation; HR, heart rate; SBP, systolic blood pressure; DBP, diastolic blood pressure; FVC, forced vital capacity; FEV_1_, forced expiratory volume in first second; WR, work rate; RER, respiratory exchange ratio; V_E_, minute ventilation; OUES, oxygen uptake efficiency slope.

* *p* < 0.05.

** *p* < 0.001.

**Figure 2:**
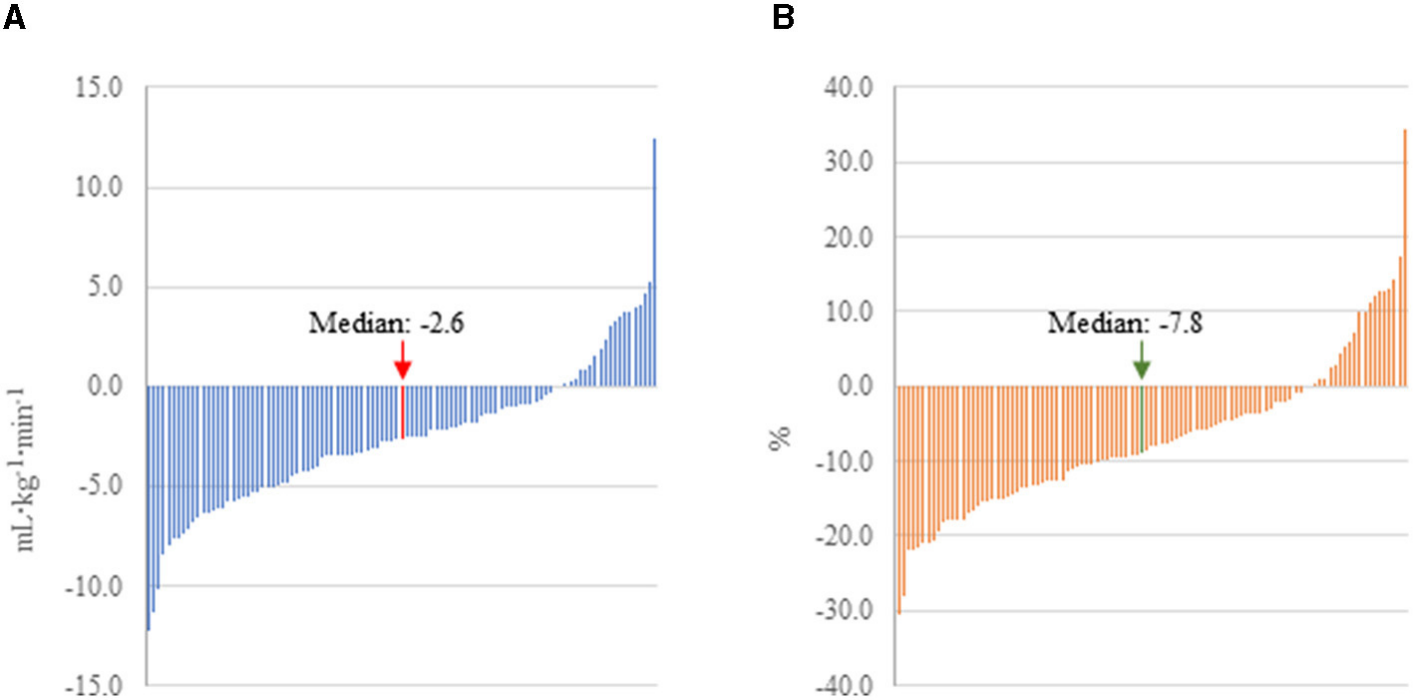
Change in peak VO_2_ pre-to post-COVID-19 in firefighters with mild to moderate illness. **(A)** CRF expressed as absolute change. **(B)** CRF expressed as percent change.

CRF was significantly lower following COVID-19 infection regardless of how it was expressed; on average, peak VO_2_ declined by 2.55 ml·kg^−1^·min^−1^ or 7.3% (*d* = −0.38, *p* < 0.001) in this cohort of firefighters. In contrast, among the subset of 70 firefighters for whom CRF data were available, peak VO_2_ did not significantly change between 2018 (34.3 ± 6.9 ml·kg^−1^·min^−1^) and 2019 (34.4 ± 6.5 ml·kg^−1^·min^−1^; *d* = 0.02, *p* = 0.766). There was considerable heterogeneity in the change in CRF following COVID-19 with some firefighters decreasing over 10 ml·kg^−1^·min^−1^ (over 20% reduction), while some firefighters had considerable improvement in their CRF following COVID-19 infection (Figure 2). Interestingly, despite the significant reduction in CRF following COVID-19 infection, changes in work exertion were limited: work efficiency significantly decreased (*d* = −0.34, *p* = 0.013), but peak WR and peak RER remained relatively stable.

Decreases in CRF could be explained by impairment in pulmonary, cardiovascular or peripheral measures. Ventilatory efficiency, assessed through the V_E_/VCO_2_ slope and OUES, decreased following COVID-19 infection (*d* = 0.39, *p* < 0.001), but no significant changes were observed in peak V_E_ and breathing reserve. Significant reductions were observed in SBP peak (*d* = −0.71, *p* < 0.001), DBP peak (*d* = −0.42, *p* < 0.001), and peak O_2_ pulse (*d* = −0.32, *p* < 0.001), but no significant changes were observed in HR peak, HR recovery, and Δ HR/WR slope. Notably, the largest post-COVID-19 change was in AT: on average, oxygen consumption at AT declined 4.27 ml·kg^−1^·min^−1^ or 24.3% (*d* = −1.12, *p* < 0.001). HR at AT also significantly declined following COVID-19 infection (*d* = −0.51, *p* < 0.001).

**Figure 3:**
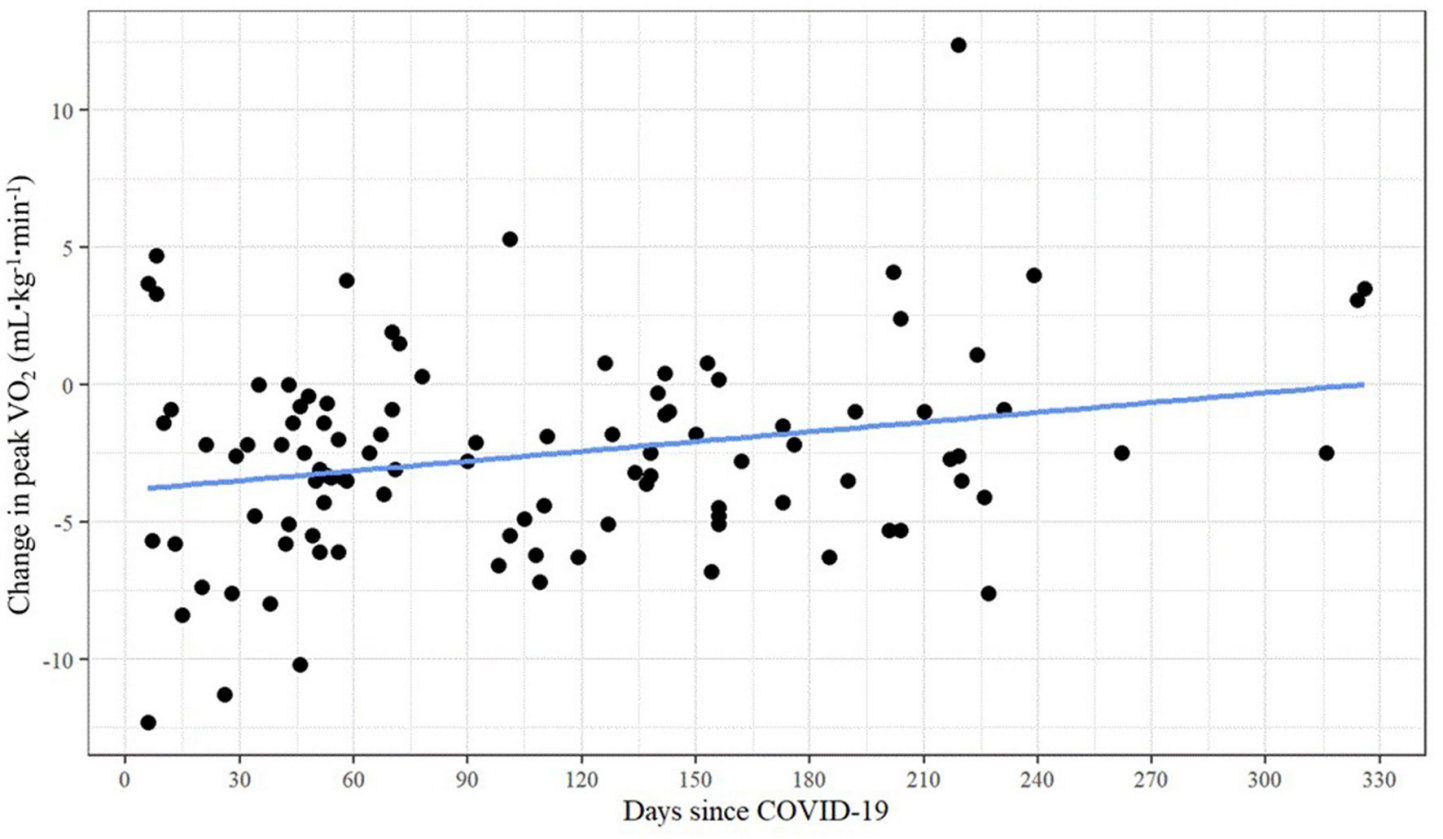
Linear relationship between change in peak VO_2_ and days since COVID-19.

As shown in Table 3, unadjusted bivariate correlations revealed a small yet significant relationship between days past COVID-19 and peak VO_2_ change (*r* = 0.250, *p* = 0.011). There was no evidence of multicollinearity between the independent variables (with all correlations | < 0.536|). Figure 3 presents the scatterplot of days since COVID-19 and change in peak VO_2_.

**Table 3:** Bivariate correlations between dependent and independent variables.

**Variables**	**1**	**2**	**3**	**4**	**5**	**6**
1. Sex	−					
2. Age	0.026	−				
3. Pre-COVID-19 peak VO_2_	0.214^*^	−0.408^**^	−			
4. BMI	0.112	0.169	−0.536^**^	−		
5. Days since COVID-19	−0.078	−0.017	−0.112	0.146	−	
6. Change in peak VO_2_	−0.006	0.069	−0.284^**^	−0.025	0.250^*^	—

* *p* < 0.050.

** *p* < 0.010.

BMI, body mass index.

**Figure 4:**
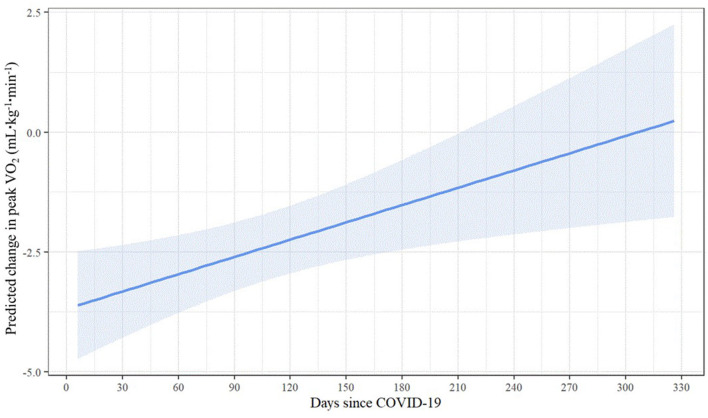
Adjusted predicted change in peak VO_2_ across elapsed COVID-19 recovery time.

Results of the linear regression model predicting change in peak VO_2_ are displayed in Table 4. Even when controlling for sex, age, BMI, and pre-COVID-19 peak VO_2_, the significant relationship between days since COVID-19 and change in peak VO_2_ persisted. Longer elapsed time was associated with smaller predicted change in peak VO_2_ [*b* = 0.01, 95% CI (0.00, 0.02), *p* = 0.007], suggesting that recovery time may have a positive incremental effect on peak VO_2_ change following COVID-19 infection. As shown by the estimated marginal effects (Figure 4), when holding the control variables constant, the predicted reduction in peak VO_2_ is less than 1 ml·kg^−1^·min^−1^ at 220 or more days post-COVID-19 and nearly zero at ~300 days post-COVID-19. Among the control variables, higher BMI [*b* = −0.31, 95% CI (−0.52, −0.10), *p* = 0.004] and pre-COVID-19 peak VO_2_ values [*b* = −0.28, 95% CI (−0.41, −0.14), *p* < 0.001] were associated with larger reductions in peak VO_2_ following COVID-19 infection.

**Table 4:** Linear regression and model predicting changes in peak VO_2_ following COVID-19.

**Variables**	***b***	***SE***	**95% CI**	***p*-value**
Male	2.11	1.26	−0.42, 4.63	0.102
Age	−0.03	0.04	−0.11, 0.05	0.428
Pre-COVID-19 peak VO_2_	−0.28	0.11	−0.41, −0.14	<0.001
BMI	−0.31	0.12	−0.52, −0.10	0.004
Days since COVID-19	0.01	0.00	0.00, 0.02	0.007

Adjusted r^2^ = 16.8%; *b*, unstandardized beta coefficient; *SE*, standard error; CI, 95% confidence interval; BMI, body mass index.

## 4 Discussion

To the best of our knowledge, this is the first study to describe longitudinal changes in CRF among an occupationally active cohort of firefighters following COVID-19 infection.

The major study finding was that CRF, specifically peak VO_2_, decreased an average of 2.6 ml·kg^−1^·min^−1^ (7.3%) in firefighters an average of 110 days past reporting mild to moderate COVID-19 infection. We also found a modest, though significant, relationship between days past COVID-19 and peak VO_2_ change (*r* = 0.250, *p* = 0.011). Reductions in CRF may impact the ability of firefighters to effectively perform their strenuous work duties and increase their health risks.

Very few studies have been able to assess longitudinal CRF changes in healthy individuals following a mild to moderate COVID-19 illness. Typically, studies have assessed CRF in hospitalized patients or those who continue to suffer from prolonged symptoms (e.g., Long COVID), usually by comparing results from testing performed during recovery to population-based norms or uninfected control groups due to a lack of a pre-COVID-19 CPET data. For example, Baratto et al. (14), found that at hospital discharge (median 30 days of hospitalization), individuals with COVID-19 had a peak VO_2_ that was 35% lower than a control group of outpatients. Debeaumont and colleagues (29) reported data indicating that CRF values remained low 6 months post hospital discharge. These studies indicate that CRF is dramatically affected by severe COVID-19 illness (to an extent that cannot be explained by bedrest alone) and that exercise tolerance remains impaired for at least 6 months among those who experienced severe COVID-19 illness and/or ongoing symptoms. Understanding longitudinal changes in CRF in occupationally active adults is important since a large majority of the population has experienced mild to moderate COVID-19 and CRF is an important and predictive health measure, particularly in firefighters who perform important public safety work.

Studies of athletes who had pre-COVID-19 CPET provided some insight into longitudinal changes in CRF for those with mild to moderate illness. Parpa and Michaelides (24) reported a 5.2% (3.01 ml·kg^−1^·min^−1^) decrease in CRF among Division 1 collegiate soccer players 60 days following mild to moderate COVID-19 illness. Similarly, Sliz et al. (25) found a 5.9% (2.84 ml·kg^−1^·min^−1^) decrease in the CRF of middle-aged (mean 40 years) professional and amateur endurance-trained athletes an average of 155 days following the end of a mild COVID-19 illness. These changes were slightly less (on a percentage basis) than the 7.3% decrease in peak VO_2_ an average of 110 days post-COVID-19 found in this study. However, when expressed in ml·kg^−1^·min^−1^, changes in peak VO_2_ were slightly higher in the athletes than among firefighters. Interestingly, the 2.84 ml·kg^−1^·min^−1^ difference in peak VO_2_ that Sliz et al. found in middle aged athletes an average of 155 days post COVID-19 was very similar to the 2.55 ml·kg^−1^·min^−1^ difference we found in middle-aged firefighters an average of 110 days post COVID-19 illness.

The only study we are aware of that investigated longitudinal changes in CRF among a work group was conducted by Štěpánek et al. (21) who investigated healthcare workers over a much longer duration than our study or the previously cited athlete studies. Repeat CPET evaluations were performed in this group an average of 762 days apart, with the second CPET occurring ~321 days after COVID-19 was reported. In this sample comprised primarily of women (95%; mean age = 55.7 years), peak VO_2_ was significantly lower in the post-COVID-19 group (13.2%, 3.12 ml·kg^−1^·min^−1^) but not significantly different between repeat measures in the control group (2.6%, 0.56 ml·kg^−1^·min^−1^). The healthcare workers in this study had a greater decrement in peak VO_2_ than firefighters in our cohort (13.2%, 3.12 ml·kg^−1^·min^−1^ vs. 7.3%, 2.55 ml·kg^−1^·min^−1^, respectively). This is somewhat surprising given that the repeat CPET evaluation in the healthcare workers was administered an average of 321 days post-COVID-19 (compared to our average of 110 days post-COVID-19) and the limited data that is available suggests that peak VO_2_ improves as time passes (19).

CPET is considered the gold standard for assessing CRF (a proposed vital sign), is important for the assessment of ischemic heart disease, and is a potential tool that could be used more effectively in clinical practice (3, 30, 31). In addition to directly measuring peak VO_2_, CPET testing also provides insights into mechanisms that explain exercise intolerance. Past studies have demonstrated multiple mechanisms for reduced CRF after COVID-19 infection including dysfunctional breathing, impaired pulmonary gas exchange (i.e. ventilation-perfusion mismatching), endothelial dysfunction, myocarditis, autonomic dysfunction, chronotropic incompetence, mitochondrial dysfunction and muscle deconditioning (16, 20, 31–38). We found that V_E_ was not different between the pre- and post-COVID-19 measurements, but V_E_/VCO_2slope_ was significantly higher and the OUES was significantly lower, consistent with normal ventilatory mechanics but impaired ventilatory efficiency (impaired pulmonary circulation or increased dead space ventilation). Although impaired ventilatory efficiency is an independent predictor of poor health outcomes and mortality in many disease states (39), the small difference we observed is of unknown clinical significance. Schwendinger et al. (19), provided a narrative review of studies using CPET to evaluate changes in CRF and mechanisms for that decline in the context of the “Wasserman gears” (40). These authors concluded that the contribution of respiratory function to the lower CRF post-COVID-19 seems to be minor, with our findings supporting that conclusion. We found that peak HR did not differ between the pre- and post-COVID-19 measurements, making chronotropic incompetence as a cause of decreased CRF less likely in this population. Both peak SBP and DBP were lower following COVID-19 infection which may be a marker of autonomic dysfunction. There was also a significant decrease in peak O_2_ pulse, which can be due to impaired peak stroke volume (endothelial dysfunction or myocarditis) or decreased peripheral skeletal muscle extraction (mitochondrial dysfunction). The reduced peak cardiac output (product of stroke volume and HR) could potentially explain the lower blood pressure at peak that was observed. The AT is the level at which oxygen consumption is above aerobic energy production and must be supplemented by anaerobic mechanisms; it is dependent on oxygen delivery to the tissues. We found that VO_2_ at AT significantly decreased by 24.3% in the firefighters following COVID-19. A decreased AT can be caused by an impaired cardiac output response during submaximal workload (suggested by the reduced work efficiency – ΔVO_2_/ΔWR) or by impaired mitochondrial function. Rinaldo et al. (41) evaluated COVID-19 patients an average of 97 days after hospital discharge and found that among those with decreased peak exercise capacity (i.e., predicted maximal VO_2_ of <85%), 37% had a reduced AT (<45%) and AT was significantly lower (*p* < 0.001) in the COVID-19 group with reduced exercise capacity (48 ± 9% of peak VO_2_ predicted) compared to normal exercise capacity (62 ± 13% of peak VO_2_).

While we found that CRF was ~7% lower following COVID-19, it is important to note the heterogeneity in the data. In fact, several firefighters in our cohort had peak VO_2_ values 20% lower (~10 ml·kg^−1^·min^−1^) than pre-COVID-19, while a few firefighters improved their CRF. Improvements may reflect an individual's personal motivation to improve their CRF and/or more time for exercise secondary to the pandemic restrictions on work and social activities. In fact, one individual increased his peak VO_2_ by over 10 ml·kg^−1^·min^−1^ (30%). It is unclear whether such a dramatic change reflected increased fitness alone or also reflects a lower than maximal performance (perhaps due to mild illness or injury) in the pre-COVID-19 test. However, this firefighter also lost a significant amount of body weight and had other positive health changes indicative of improved fitness. The decrease in CRF (7.3%) that we observed between annual health exams cannot be explained by age-related changes. In general, estimated peak VO_2_ is reported to decline at a rate of ~10% per decade (~1%/year) (42, 43). Further, we evaluated CRF data from 2018 and compared it to 2019 data (available for a subset of 70 firefighters) and found no significant decrease in CRF. In our study, higher BMI was associated with larger reductions in CRF. While we do not have data to explore the mechanisms responsible for this association, previous literature has shown that those with obesity were at higher risk of experiencing worse cases of COVID-19, which could explain this finding (44).

Understanding the time course of recovery for CRF has been difficult based on a lack of published studies and a variety of methodologies. However, due to variability in when the firefighters in our study received their CPET and experienced COVID-19, we were able to investigate the impact of time since COVID-19 infection on CRF. Our results suggest that increasing time from COVID-19 resulted in a small, yet beneficial, effect on change in peak VO_2_. By 300 days post-COVID-19, the predicted reduction in peak VO_2_ was nearly zero when control variables were held constant. In their narrative review, Schwendinger et al. (19) reported that percent-predicted peak VO_2_ was dramatically reduced in studies that evaluated short term changes (within 30 days of diagnosis) and that CRF was similarly low (~85% of percent-predicted maximal VO_2_) in studies that reported results in the moderate term (1–5 months post-COVID-19) and long-term (>5 months post-COVID-19). Cassar et al. (15) performed serial cardiac and lung magnetic resonance imaging and CPET to describe the natural history of recovery at 2–3 and 6 months post-COVID-19 in a group of previously hospitalized patients (mean age 55 years) who were experiencing persistent symptoms and compared results to a control group. At the 2–3 month post-COVID-19 assessment, the control group had a significantly higher CRF than the patients (peak VO_2_ 28.1 and 18.0 ml·kg^−1^·min^−1^, respectively). At 6 months post-COVID-19, the patients had improved their peak VO_2_ (20.5 ml·kg^−1^·min^−1^) but remained significantly lower than the control group. While these subjects were older (and hence had lower peak VO_2_) and sicker (they were hospitalized with COVID-19) than our cohort, the gradual improvement in CRF over many months is consistent with our findings.

Our findings support the general understanding that CRF recovers over time, but also reveals important novel findings, namely that CRF was significantly and meaningfully reduced following mild to moderate COVID-19 infection and that recovery took several months in a substantial portion of the cohort. It is unknown if the COVID-19 related decrease in peak VO_2_ is more pronounced than what might result from other types of respiratory infections, but as the majority of firefighters have been infected with SARS-CoV-2, this a concerning issue.

Our findings are especially relevant for occupational cohorts who must perform strenuous work. Further complicating the issue of Fitness for Duty in public safety professions is the fact that reductions in CRF may not be appreciated by the individual. This suggests that occupational physicians and other healthcare providers who work with firefighters (and other occupational cohorts) should monitor fitness to support individuals who may have experienced significant CRF reductions following COVID-19 infection. The data also suggests that training instructors and incident commanders should consider that the same amount of work output may not be possible following COVID-19 illness.

### 4.1 Strengths and limitations

These findings must be viewed in light of the study's limitations. First, COVID-19 date of diagnosis, testing, and symptom onset were self-reported by firefighters, which introduces the possibility of recall errors. In addition, this study defined severe illness as requiring hospitalization, leaving a wide range of COVID-19 illness severity from mild to moderate. As reflected by the wider confidence intervals, there was also less precision in predicted change at higher values of elapsed time since COVID-19 given the distribution of the sample. Data for this study were captured during the earliest part of the COVID-19 pandemic when vaccinations were unavailable and early virus strains were circulating, which limits generalizability to other time periods. And, although consistent with the makeup of the fire service, females were under-represented in the current cohort. Despite noted limitations, a particular strength of this study is access to a cohort of occupationally active, middle-aged individuals in public safety who had annual medical exams that included CPET both prior to and after having COVID-19 allowing longitudinal assessment of individuals. In addition, because there was variability in when firefighters had their annual exams and when they experienced COVID-19, this study was able to investigate the impact of time since COVID-19 on CRF, a design not employed by most other studies that administered CPET at specified time points post-COVID-19.

## 5 Conclusion

We observed a 7.3% reduction in the directly measured CRF of occupationally active firefighters who reported for their scheduled annual health exam after having had COVID-19. This reduction remained significant even when controlling for sex, age, BMI, and pre-COVID-19 peak VO_2_. Although predicted values based on regression analysis indicate that most individuals returned to pre-COVID-19 CRF values by ~300 days, there is considerable variability in our observed data with some individuals experiencing substantially reduced CRF many months after COVID-19 infection. It is unclear if the reductions in CRF are due entirely to viral infection (and a broad range of organ disruption that can occur secondary to infection) or if altered activity patterns contributed to lower fitness. Regardless of the reason, lower CRF is of concern for firefighters (and other public safety personnel) who may be called on to perform strenuous work. Additional research is needed to understand more fully how CRF recovers in different groups (e.g., those who have had mild, moderate, and/or severe COVID-19 *and* been vaccinated; those who have endured multiple bouts of COVID-19; those who experienced or are experiencing Long COVID, etc.), the mechanisms responsible for impaired exercise tolerance (i.e., mitochondrial dysfunction, endothelial dysfunction, autonomic nervous system imbalances), and whether targeted interventions may help to more quickly restore pre-COVID-19 CRF.

## Data availability statement

The original contributions presented in the study are included in the article/supplementary material, further inquiries can be directed to the corresponding author.

## Ethics statement

The study involving humans was approved by Skidmore College Institutional Review Board. The study was conducted in accordance with the local legislation and institutional requirements. Written informed consent for participation was not required from the participants or the participants' legal guardians/next of kin in accordance with the national legislation and institutional requirements.

## Author contributions

SD'I: Conceptualization, Writing—original draft, Writing—review & editing, Data curation, Project administration. LB: Formal analysis, Writing—review & editing. AF: Writing—review & editing. CT: Writing—review & editing. KJ: Data curation, Writing—review & editing. SC: Data curation, Writing—review & editing. RA: Writing—review & editing. DS: Conceptualization, Funding acquisition, Methodology, Supervision, Writing—original draft, Writing—review & editing, Resources.
